# High-Protein Supplementation and Neuromuscular Electric Stimulation after Aneurysmal Subarachnoid Hemorrhage Increases Systemic Amino Acid and Oxidative Metabolism: A Plasma Metabolomics Approach

**DOI:** 10.21203/rs.3.rs-3600439/v1

**Published:** 2023-11-17

**Authors:** Aaron M Gusdon, Jude PJ Savarraj, Dians Feng, Adam Starkman, Guoyan Li, Uttam Bodanapally, William Denny Zimmerman, Alice S Ryan, Huimahn A Choi, Neeraj Badjatia

**Affiliations:** University of Texas John P and Katherine G McGovern Medical School; University of Texas John P and Katherine G McGovern Medical School: The University of Texas Health Science Center at Houston John P and Katherine G McGovern Medical School; University of Texas John P and Katherine G McGovern Medical School: The University of Texas Health Science Center at Houston John P and Katherine G McGovern Medical School; University of Maryland Hospital: University of Maryland Medical Center; University of Maryland Hospital: University of Maryland Medical Center; University of Maryland Hospital: University of Maryland Medical Center; University of Maryland Hospital: University of Maryland Medical Center; University of Maryland Hospital: University of Maryland Medical Center; University of Texas John P and Katherine G McGovern Medical School: The University of Texas Health Science Center at Houston John P and Katherine G McGovern Medical School; University of Maryland Hospital: University of Maryland Medical Center

**Keywords:** Aneurysmal subarachnoid hemorrhage, high protein diet, neuromuscular electrical stimulation, metabolomics, N-acetylleucine, quinolinate

## Abstract

**Background:**

The INSPIRE randomized clinical trial demonstrated that a high protein diet (HPRO) combined with neuromuscular electrical stimulation (NMES) attenuates muscle atrophy and may improve functional outcomes after aSAH. Using an untargeted metabolomics approach, we sought to identify specific metabolites mediating these effects.

**Methods:**

Blood samples were collected from subjects on admission prior to randomization to either standard of care (SOC; N=12) or HPRO+NMES (N=12) and at 7 days as part of the INSPIRE protocol. Untargeted metabolomics were performed for each plasma sample. Paired fold changes were calculated for each metabolite among subjects in the HPRO+NMES group at baseline and 7 days after intervention. Changes in metabolites from baseline to 7 days were compared for the HPRO+NMES and SOC groups. Sparse partial least squared discriminant analysis (sPLS-DA) identified metabolites discriminating each group. Pearson’s correlation coefficients were calculated between each metabolite and total protein per day, nitrogen balance, and muscle volume Multivariable models were developed to determine associations between each metabolite and muscle volume.

**Results:**

A total of 18 unique metabolites were identified including pre and post treatment and differentiating SOC vs HPRO+NMES. Of these, 9 had significant positive correlations with protein intake: N-acetylserine (ρ=0.61, *P*=1.56×10^−3^), N-acetylleucine (ρ=0.58, *P*=2.97×10^−3^), β-hydroxyisovaleroylcarnitine (ρ=0.53, *P*=8.35×10^−3^), tiglyl carnitine (ρ=0.48, *P*=0.0168), N-acetylisoleucine (ρ=0.48, *P*=0.0183), N-acetylthreonine (ρ=0.47, *P*=0.0218), N-acetylkynurenine (ρ=0.45, *P*=0.0263), N-acetylvaline (ρ=0.44, *P*=0.0306), and urea (ρ=0.43, *P*=0.0381). In multivariable regression models, N-acetylleucine was significantly associated with preserved temporalis [OR 1.08 (95%CI 1.01, 1.16)] and quadricep [OR 1.08 (95%CI 1.02, 1.15)] muscle volume. Quinolinate was also significantly associated with preserved temporalis [OR 1.05 (95%CI 1.01, 1.09)] and quadricep [OR 1.04 (95%CI 1.00, 1.07)] muscle volume. N-acetylserine, N-acetylcitrulline, and b-hydroxyisovaleroylcarnitine were also associated with preserved temporalis or quadricep volume.

**Conclusions:**

Metabolites defining the HPRO+NMES intervention mainly consisted of amino acid derivatives. These metabolites had strong correlations with protein intake and were associated with preserved muscle volume.

## Introduction

Aneurysmal rupture causing subarachnoid hemorrhage (aSAH) accounts for 5–10% of all strokes in the United States but often affects younger patients contributing to significant morbidity and mortality^1,2^. Despite an improved understanding of the pathophysiology of aSAH effective treatments have remained elusive^3^.

Increased systemic breakdown of protein following injury has long been recognized to be associated with worse outcomes^4–6^. These findings have led to recommendations for increased protein (1.2–2.2 g/kg) to be delivered to patients following traumatic injury^7^. However, the benefits of a higher protein diet in critically ill patients remains to be proven and must be used cautiously in patients with acute kidney injury^8^.

Similar to patients with a traumatic injury, aSAH results in a systemic catabolic state due to increased catecholamine release and cytokine production^9^. Immune-mediated malnutrition (IMM) characterized after SAH is characterized by a pro-inflammatory hypermetabolic state coupled with protein energy catabolism^10–12^. Specifically, acute reductions of glutamine, an amino acid essential in maintaining muscle mass, has been closely linked to the sequelae of malnutrition in critical illness^13^ and long-term recovery after SAH^10^. Muscle weakness and impaired neuromotor recovery can occur if nutrition delivered curing the critical illness phase after aSAH is inadequate to compensate for increased catabolism^10,14^ The recent Impact of neuromuscular electrical stimulation (NMES) and high protein supplementation (HPRO) on Recovery After SAH (INSPIRE) trial demonstrated that a HPRO diet combined with NMES decreases muscle wasting after aSAH and contributes to improved functional outcomes^15^. However, the specific protein metabolites and molecular mechanisms subserving this benefit remain unclear.

Herein, we utilized an untargeted metabolomics approach to evaluate circulating metabolites in patients enrolled in the INSPIRE clinical trial who received either standard of care (SOC) or HPRO + NMES. We sought to evaluate whether specific metabolites of amino acid metabolism are elevated after HPRO intervention and to determine which metabolites may mitigate muscle wasting after aSAH.

## Methods

### Subjects.

Subjects were selected among the 25 enrolled in the INSPIRE phase 2 randomized controlled trial (NCT03201094) who had available plasma samples^15^. Detailed methods are available in the published trial results^15^. All patients enrolled in INSPIRE had a diagnosis of aSAH, underwent aneurysm repair within 48 hours of ictal hemorrhage, were at least 18 years of age, and had a Hunt Hess score (HHS) ≥ 2 and modified Fisher Scale (mFS) score > 1. Patients with subarachnoid hemorrhage for etiologies other than a ruptured aneurysm (trauma, arteriovenous malformation, neoplasm) were excluded. Patients were excluded if they were unlikely to survive one-week post hemorrhage or unlikely to remain in the ICU for more than 7 days. Patients with a body mass index (BMI) < 15 or > 40 kg/m^2^, protein allergy, premorbid modified Rankin Scale (mRS) score > 1, or who were currently pregnant or diagnosed with a malignancy, inflammatory disorder, neuromuscular disorder, or renal failure were also excluded.

All subjects were randomized to either standard of care (SOC) or high protein supplementation and neuromuscular electrical stimulation (HPRO + NMES). Subjects in the HPRO + NMES group were administered a bolus of a whey protein power dissolved in water (8–10 ounces) three times daily with a dose of 3 g leucine/feeding for a goal of 1.75 g/kg/d. SOC subjects received 1.2–1.4 g/kg/day of protein delivered via enteral nutritional formulas or specific oral diets with no additional protein supplementation permitted. The NMES device was a L300 Plus^®^ system (Bioness, Inc, Valencia, CA) with thigh cuffs applied bilaterally to stimulate with stimulator pads across the quadricep muscles. The NMES intervention included two 30-minute session per day. All subjects underwent study interventions until post-bleed day (PBD) 14. Each subject was followed up at 90-days in person for outcome measurements. The Short Performance Physical Battery (SPPB) was used to measure physical recovery. The SPPB assesses lower extremity mobility including gait speed, standing balance, and lower extremity strength and endurance^16^.

### Biosamples.

Blood samples were collected from subjects within 24-hours of admission (before randomization to SOC or HPRO + NMES) and at PBD 7 in ethylenediaminetetraacetic acid (EDTA) containing tubes. Samples were centrifuged at 4°C and stored at −80°C until analysis. A total of 12 subjects from the SOC group and 12 subjects from the HPRO + NMES group had plasma samples available at both time points. The study and use of biosamples complies with ethical standards, and institutional review board (IRB) approval was obtained for all studies (HSC-MS-12-0637).

### Metabolomics.

Plasma samples (200 μL) were sent to Metabolon (Durham, NC, USA) for untargeted metabolomics analysis in a single batch. Detailed descriptions of the metabolomics platform, which consists of four independent ultra-high-performance liquid chromatography-tandem mass spectrometry (UPLC-MS/MS) instruments and methods have been published elsewhere^17–19^. Median and standard deviation of internal standards are used to assess instrument variability. Identification of each metabolite was accomplished by automated comparison of each ion to a standard library. Areas of under the curve (AUC) were calculated for each peak. Raw AUC values were normalized correcting for between day variation in instrument calibration using internal standards and median value for each run day. Missing values were imputed using k-nearest neighbors with 10 neighbors used for each imputation. All results were subsequently log transformed.

### Image acquisition.

Included patients were scanned using one of two scanners: a 64-section CT unit (Brilliance; Philips Healthcare, Andover, MA) or a 128-section dual-source CT unit (SOMATOM Force; Siemens, Erlangen Germany). Images were archived at 3 mm section thickness. Baseline and follow-up series also were saved as Neuroimaging Informatics Technology Initiative (NifTI) files for voxel-wise labeling and volumetric analysis using 3D slicer (version 4.11.20210226, slicer.org).

### Image Analysis and temporal muscle volume measurement.

Temporal muscle volumes were measured from 120 kV images using label masks of the temporal muscle that was created using a 3D threshold paint tool initially in the axial plane and editing in coronal and sagittal planes. Thresholding was set to a range of 30 to 80 units to minimize noise and avoid neighboring hyperdense blood and calvarium. Labeling was performed with small (1–3 mm diameter) spherical ROI to carefully delineate the interface of the membrane with adjacent structures. Labeling was performed by a resident and all scans were subsequently reviewed and edited by a radiology attending with 14 years of experience. Both reviewers were blinded to clinical data. Muscle volume was measured from the origin to the coronoid process of mandible (first slice depicting the coronoid process). Quantitative values (milliliters) were derived for each label class using the Segment Statistics slicer module.

### Bioinformatics.

All bioinformatics analyses were performed in R (R Foundation for Statistical Computing). Fold changes (FC) were calculated for each metabolite comparing either late (7-days) vs early (within 24h of admission) or between HPRO + NMES and SOC at day 7. Changes in metabolites were considered to be significant increased at FC > 2 and decreased at FC < 2 with false discovered rate (FDR) corrected *P*-values < 0.05. Sparse partial least squared discriminant analysis (sPLS-DA) using the mixOmics library in R (http://mixomics.org). Adding discriminant analysis to the PLS algorithm allows for classification of large datasets^20–22^. We used sPLS-DA to select the most discriminative metabolites to classify groups. The *perf* function was used to determine the number of components The number of components to use in order to maintain a classification error rate less than 0.1 was determined using the *perf* function. The *tune.splsda* function was used to determine the number of metabolites in order to minimize the balanced error rate.

### Statistics.

All statistical analyses were performed in R (R Foundation for Statistical Computing). Demographic variables were compared using a *t*-test, Mann-Whitney U test, or χ-squared test as appropriate. Pearson’s correlation coefficients were calculated between each metabolite and protein per day as well as nitrogen balance. Multivariable linear regression models were developed evaluate the relationship between selected metabolites (change in levels from admission to 7 days) and muscle volume (temporalis and quadricep muscles). All models were adjusted for age, sex, and aSAH severity (HHS < 4 vs ≥ 4).

## Results

### Demographics.

Demographics for all subjects are shown in Table 1. A total of 24 subjects were included with 12 in both the SOC and HPRO + NMES groups. The groups were well matched with no significant differences in age, sex, race, HHS, DCI, mRS at discharge, and mRS at 3-months between the groups.

### Metabolites detected.

A total of 1,370 metabolites were detected from plasma samples with 261 of these metabolites unable to be identified. These metabolites were excluded resulting in a total of 1,109 metabolites available for analysis. Metabolites consisted of amino acids, carbohydrates, cofactors and vitamins, energy related pathways (glycolysis, gluconeogenesis, and TCD cycle), lipids, nucleotides, peptides, as well as partially characterized molecules (Supplementary Fig. 1).

#### Metabolite fold changes

**Figure 1:**
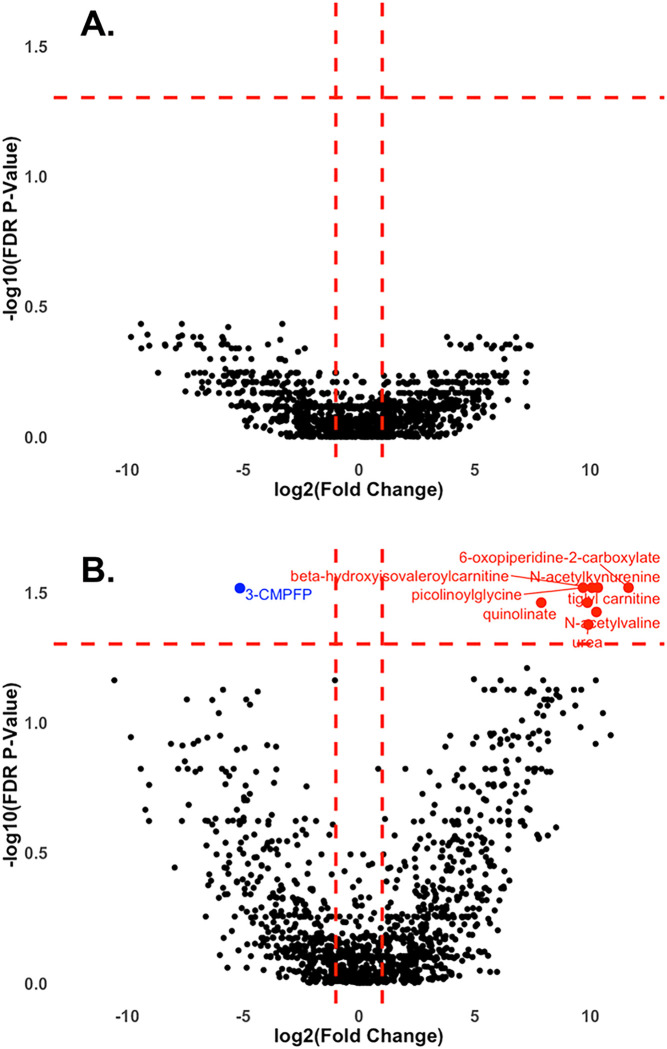
Volcano plots comparing paired metabolites at early and late time points. Paired fold changes were calculated for each subject in the SOC group (A) and HPRO+NEMS group comparing the early and 7-day timepoints. Volcano plots were created using log_2_(fold changes) and −log_10_(FDR corrected *P*-Values). Changes were considered to be significant if log_2_(fold change) was greater than 2 or less than −2 and FDR corrected *P*-value was < 0.05. Metabolite that significantly increased from admission to 7-day are shown in red, and those that decreased are shown in blue. Abbreviations: SOC (standard of care), HPRO+NMES (high protein plus neuromuscular electrical stimulation), FDR (false discovery rate), 3-CMPFP (3-Carboxy-4-methyl-5-propyl-2-furanpropionate3-Carboxy-4-methyl-5-propyl-2-furanpropionate).

Paired fold changes and corresponding FDR corrected *P*-values were calculated for each metabolite. Figure 1 shows paired fold changes between metabolite levels measured at PBD 7 compared to metabolite levels measures within 24 hours of ictus (before randomization) in the SOC group (Fig. 1A), as well as those in the HPRO + NMES group (Fig. 1B). Data are displayed as volcano plots with metabolites considered to be significantly different at a log2(fold change) of 2 or −2 and FDR P-value < 0.05. No metabolite fold changes were significantly different in the SOC group, while 8 metabolites were significantly increased and 1 significantly decreased in the HPRO + NMES group. All differentially expressed metabolites are shown in Supplementary Table 1.

#### sPLS-DA analysis

**Figure 2:**
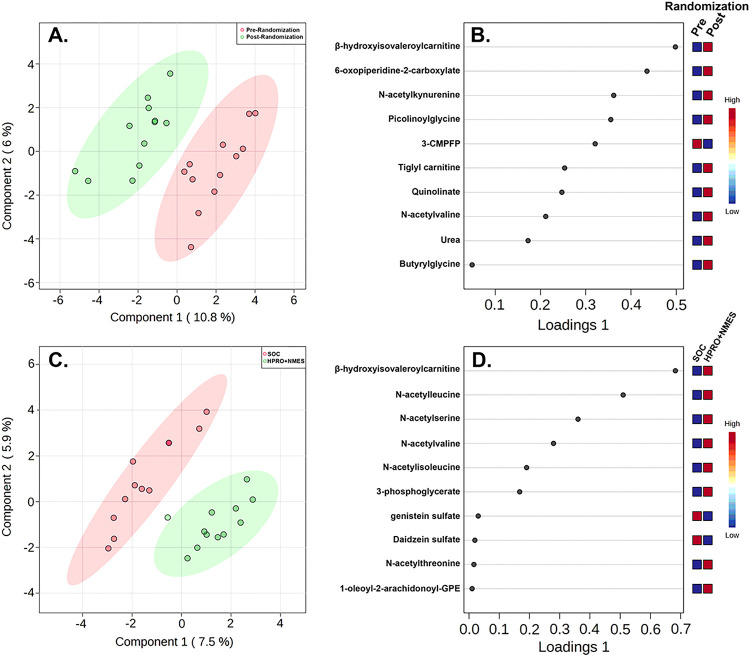
Sparse partial least squared discriminant analysis (sPLS-DA). sPLS-DA was performed to determine metabolites driving differences between groups. Groups consisted of paired changes in metabolites for subjects in the HPRO+NMES group from baseline to 7-days (A, B) and SOC vs HPRO+NMES (C, D). Loadings plots detail the top 10 metabolites accounting for differences between groups.

sPLS-DA analysis was performed to determine metabolites contributing to differences between the HPRO + NMES group pre and post randomization (Fig. 2A). The top 10 metabolites that differentiated the HPRO + NMES group pre and post randomization are shown in Fig. 2B and are also listed in Supplementary Table 2. We also determined changes in each metabolite from baseline to 7-days comparing patients in both SOC and HPRO + NMES groups. sPLS-DA was performed comparing SOC and HPRO + NMES groups using changes in each metabolite (Fig. 2C). The top 10 metabolites distinguishing SOC from HPRO + NMES are shown in Fig. 2D and also listed in Supplementary Table 2. Metabolites identified to have significant changes attributable to HPRO + NMES protocol were often amino acid metabolic intermediates. Considering both comparisons (Fig. 2B,D), a total of 18 unique metabolites were identified. Changes from baseline to 7 days in each of the 18 metabolites comparing SOC and HPRO + NMES are shown in Supplementary Fig. 2.

#### Correlations between metabolites and protein intake

In order to determine the effect of treatment on each of the 18 metabolites above, correlation coefficients were determined between changes in each metabolite from baseline to 7-days and protein intake per day considering all subjects (SOC and HPRO + NMES). As expected, there was a significant correlation between protein per day and daily nitrogen balance (ρ = 0.72, *P* = 9.57×10^− 5^). Significant correlations were also detected between protein intake and 9 of the 18 metabolites detected from the sPLS-DA analysis from Fig. 2: N-acetylserine (ρ = 0.61, *P* = 1.56×10^− 3^), N-acetylleucine (ρ = 0.58, *P* = 2.97×10^− 3^), β-hydroxyisovaleroylcarnitine (ρ = 0.53, *P* = 8.35×10^− 3^), tiglyl carnitine (ρ = 0.48, *P* = 0.0168), N-acetylisoleucine (ρ = 0.48, *P* = 0.0183), N-acetylthreonine (ρ = 0.47, *P* = 0.0218), N-acetylkynurenine (ρ = 0.45, *P* = 0.0263), N-acetylvaline (ρ = 0.44, *P* = 0.0306), and urea (ρ = 0.43, *P* = 0.0381). Correlations between protein per day intake as well as nitrogen balance and all 21 metabolites are shown in Supplementary Table 3.

Metabolites most negatively correlated with protein intake were long chain fatty acid derivates and ketones. Metabolites with correlation coefficients < −0.50 included: hexadecenedioate (C16:1) (ρ= −0.66, *P* = 4.71×10^− 4^), tetradecadienedioate (C14:2) (ρ= −0.65, *P* = 6.67×10^− 4^), 3-hydroxydodecanedioate (ρ= −0.60, *P* = 0.0021), 3-hydroxybutyrate (ρ= −0.58, *P* = 0.0032), acetoacetate (ρ= −0.57, *P* = 0.0038), tetradecanedioate (C14) (ρ= −0.53, *P* = 0.0071), octadecanedioate (C18) (ρ= −0.53, *P* = 0.0082), hexadecanedioate (C16) (ρ= −0.52, *P* = 0.0089), and dodecanedioate (C12) (ρ= −0.51, *P* = 0.011).

#### Metabolites and muscle volume

**Figure 3:**
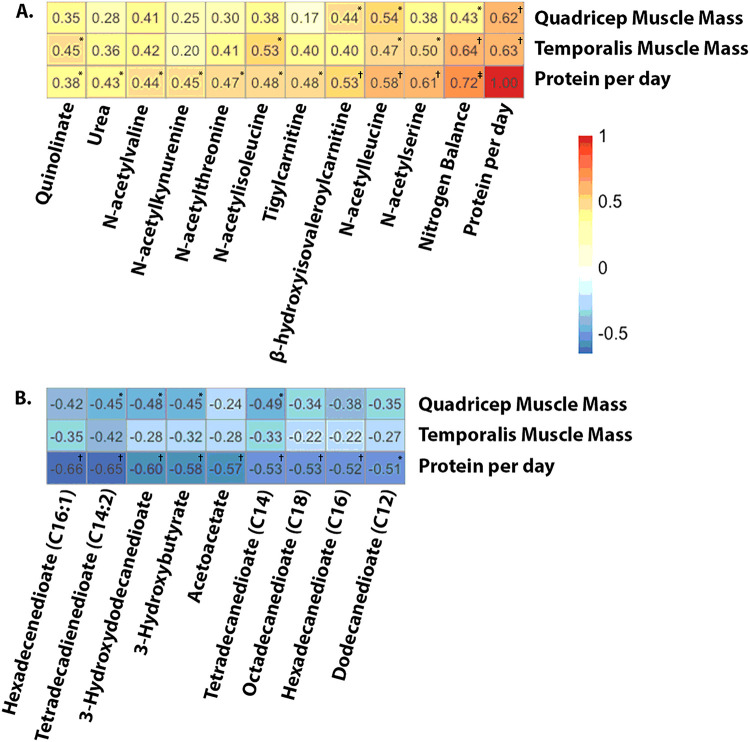
Heatmaps demonstrating correlations between metabolites and muscle volume. Pearson’s correlations coefficients were calculated between each metabolite and quadricep muscle, temporalis muscle, and protein per day (g/kg/d). Correlations were determined using changes in metabolites from baseline to 7-days. Metabolites are arranged by those having the most positive (A) and most negative (B) correlations with protein per day. Significance: *P<0.05, ^†^*P*<0.01, ^‡^*P*<0.0001

Total daily protein intake is strongly correlated with increased muscle volume in both the quadricep and temporalis muscles (ρ = 0.63, *P* = 0.0015 and ρ = 0.62, *P* = 0.002, respectively). Similarly, nitrogen balance was significantly positively correlated with increased quadricep and temporalis muscle volume (ρ = 0.43, *P* = 0.044 and ρ = 0.64, *P* = 0.0012, respectively). Increased temporal muscle volume was correlated with increased levels of N-acetylisoleucine (ρ = 0.53, *P* = 0.012), N-acetylserine (ρ = 0.50, *P* = 0.017), N-acetylleucine (ρ = 0.47, *P* = 0.027), and quinolinate (ρ = 0.45, *P* = 0.037). Increased quadricep muscle volume was correlated with increased levels of N-acetylleucine (ρ = 0.54, *P* = 0.010) and β-hydroxyisovaleroylcarnitine (ρ = 0.44, *P* = 0.038). Heatmaps depicting significant correlations between metabolites and increased muscle volume are shown in Fig. 3A. Metabolites included in Fig. 3A all have significant positive correlations with protein intake per day. Figure 3B includes those metabolites with the most negative correlations with protein intake per day and depicts their correlations with quadricep and temporalis muscle volume. Correlations between each of the 18 metabolites and temporalis and quadricep muscle preservation as well as those with significant negative correlations are shown in Supplementary Table 4.

#### Multivariable models for muscle volume

Separate multivariable models were developed to assess the association between individual metabolites and temporalis and quadricep muscle volume (Table 2). Models were developed for each of the 18 metabolites, with those models discussed below having significant associations with either temporalis or quadricep volume. Each model was adjusted for age, sex, and aSAH severity (HHS I-II vs IV-V). In the first model, the amount of protein intake per day was associated with increased volume of both temporalis [OR 1.13 (95%CI 1.07, 1.20), *P* = 5.69×10^− 4^] and quadricep [OR 1.13 (1.07, 1.19), *P* = 2.21×10^− 4^] muscles. Similarly, in the second model, higher nitrogen balance was associated with increased volume of both temporalis [OR 1.01 (1.00, 1.02), *P* = 0.0019] and quadricep [OR 1.01(1.00, 1.01), *P* = 0.045] muscles. In the third model, larger increases in N-acetylleucine were associated with increased volume of both temporalis [1.08 (1.01, 1.16), *P* = 0.038] and quadricep [1.08 (1.02, 1.15), *P* = 0.012] muscles. In the fourth model, larger increases in quinolinate were associated with increased volume of both temporalis [OR 1.05 (95%CI 1.01, 1.09), *P* = 0.018] and quadricep [OR 1.05 (95%CI 1.01, 1.09), *P* = 0.040] muscles. Larger increases in N-acetylserine [model 5: OR 1.18 (95%CI 1.04, 1.34), *P* = 0.017] and N-acetylisoleucine [model 6: OR 1.11 (1.01, 1.22), *P* = 0.048] were associated with increased temporalis muscle volume. Larger increases in β-hydroxyisovaleroylcarnitine [model 7: OR 1.07 (95%CI 1.01, 1.13), *P* = 0.026] were associated with increased quadricep muscle volume.

## Discussion

The INSPIRE trial demonstrated that a high protein diet delivered enterally combined with neuromuscular electrical stimulation reduces muscle atrophy in the quadricep muscle after aSAH^15^. Herein, we identify the effects of HPRO + NMES on circulating plasma metabolites and examine the associations between changes in metabolite levels and muscle volume in both quadricep and temporalis muscles.

Using untargeted metabolomics, we identified the effects of a combined treatment (HPRO + NMES) on metabolite concentrations. Specifically, 18 metabolites that were prominently affected by the treatment. Our findings support the concept that nutritional and muscle stimulation interventions change the metabolome and these changes can contribute to overall muscle preservation in the acute stages of aSAH. Not surprisingly, among HPRO + NMES patients, there were notable increases in amino acid derivatives in plasma. Many of these amino acid derivatives, especially as related to glutamine metabolism are established important building blocks to preserving muscle mass. We also found a carnitine ester (β-hydroxyisovaleroylcarnitine, tigylcarnitine), a tryptophan catabolite (quinolinate), a delta-lactam (6-oxopiperidine-2-carboxylate), a phosphatidylethanolamine (1-oleoyl-2-arachidonoyl-GPE (18:1/20:4), a monophosphoglycerate involved in glycolysis and the calvin cycle (3-phosphoglycerate), and urea to be higher after HPRO + NMES treatment. Other metabolites including the isoflavones genistein and daidzein were lower in the HPRO + NEMS group. These metabolites are present in their sulfonic acid conjugates, which is their most common circulating form after undergoing hepatic metabolism by sulfotransferase enzyme^23^. The furoic acid 3-carboxy-4-methyl-5-pentyl-2-furanpropionate (3-CMPFP) was also decreased in the HPRO + NMES group.

Our analysis showed that as expected, half of the 18 that were affected by the treatment were significantly positively correlated with protein intake per day (Supplementary Table 3), namely the acetylated amino acids as well as other metabolites, such as the carnitine ester β-hydroxyisovalerocylcarnitine, which plays a role in leucine catabolism. Urea, the end product of the urea cycle that plays a pivotal role in metabolizing excess nitrogen was also correlated with increased protein intake per day^24^. Additionally, an acylcarnitine (tiglylcarnitine) was correlated with protein intake per day. Although not a direct amino acid derivative, tiglyl containing compounds play a role in the metabolism of isoleucine^25^. Consistently, metabolites that were most negatively correlated with protein intake per day consisted of fatty acids and ketones (Supplementary Table 3) suggesting a shift away from fatty acid and ketone metabolism in those subjects receiving more dietary protein. While a shift away from fatty acid metabolism may attenuate inflammation^26^, some fatty acids such as omega-3 polyunsaturated fatty acids as well as ketones are thought to play a beneficial role^27^. Future studies will be necessary to understand the functional significance of decreased fatty acids and ketones on functional recovery.

In our previous study, atrophy of the quadricep muscle correlated with protein intake. In this analysis, we add to our findings by confirming in a multivariable model adjusting for age, sex and aSAH severity that that higher protein intake per day as well as higher nitrogen balance were associated with not only perseveration of the quadricep muscle but temporalis muscle as well. Recent reports indicate that differences in temporalis muscle volume may be a marker of disease severity and prognosis after aSAH^28^. Our findings would suggest that this muscle may also be a sensitive marker of nutritionally-driven metabolomic changes after aSAH.

Increased levels the acylated amino acid N-acetylleucine were associated with both temporalis and quadricep muscles preservation, while other acetylated amino acids were only significantly associated with temporalis muscle preservation. Although the quadricep muscle was exposed to facilitated exercise with NMES during the study period, the smaller size of the temporalis may have made smaller changes in muscle mass easier to detect. The BCAA leucine and its metabolic derivatives have increase in paradigms of amino acid supplementation combined with exercise. Leucine in particular impacts the mTORC1 pathway and plays an integral role in energy homeostasis^29^. N-acetylleucine (NAL) has therapeutic potential, with studies showing that it may improve motor function in cerebral ataxia^30^ as well as lysosomal storage disease including Niemann Pick^31,32^ and GM2 Gangliosidosis^33,34^. More recently, NAL treatment has been shown to improve motor and cognitive outcomes after TBI in a mouse model^35^. NAL therefore may be a promising treatment for functional recovery after aSAH.

Increased levels of quinolinate were also shown to be associated with muscle preservation. Quinolinate plays an important role in tryptophan metabolism via the kynurenine pathway^36,37^. While upstream metabolites such as kynurenine have been associated with muscle wasting during critical illness^38,39^, quinolinate does not have this effect^40^. Quinolinate can be used to synthesize nicotinamide adenine dinucleotide (NAD^+^), which is depleted in response to proinflammatory stimuli^41^. Numerous reactions rely on NAD^+^ such as DNA repair via poly-ADP ribosylation and sirtuins, which have regulatory roles in cellular metabolism^42,43^. Although it is unclear whether quinolinate has a benefit on functional outcomes after aSAH, it is possible that the higher protein intake provides more tryptophan as a substrate to produce quinolinate resulting in the ability to restore NAD^+^ depleted after aSAH and the subsequent robust inflammatory response. Although we measured circulating quinolinate in plasma, quinolinate is also well known to have neurotoxic effects in the CNS as it is an agonist of the N-methyl-D-aspartate (NMDA) receptor and acts as an excitotoxin^44^. Therefore, additional studies will be needed to determine whether circulating quinolinate plays a beneficial role or is primarily a bioproduct of higher protein intake.

High protein diets have been extensively evaluated in critically ill patients. Data from patients with traumatic brain injury (TBI) have suggested that a higher protein intake (1.5–2.0 g/kg/day) may be beneficial for recovery^45^. However, the recent EFFORT Protein trial found no significant survival benefit of a high protein diet alone with an increased risk of acute kidney injury (AKI) in at risk patients^8^. This large, randomized trial was conducted in mechanically ventilated patients without acquired brain injury, limiting its applicability to our study. In this study, we have identified specific metabolites that are increased by HPRO + NMES treatment which in turn were also associated with intermediaries of energy homeostasis and muscle preservation. This suggests that a more tailored intervention including a combination of metabolites such as acetylated amino acids like N-acetylleucine along with facilitated exercise with NMES may be able to preserve muscle mass while avoiding the potentially deleterious effects of a high protein diet.

This study has several important limitations. First, the small sample size (12 subjects in each group) as well as numerous comparisons being made for a broad panel of metabolites may have resulted in some associations been found by random chance. We attempted to mitigate this by utilizing false discovery rate corrected *P*-values. This small samples size also precluded being able to make associations with functional outcomes. Second, patients in the intervention arm received a combination of HPRO and NMES making it difficult to deconvolute the effects of these two interventions. Although we determined associations between metabolites and protein per day, overlapping effects of NMES may have contributed to some of the changes in metabolites observed. Third, this study was conducted at a single tertiary care facility, therefore given variability in practices and patient populations, these results may not be broadly generalizable. Finally, although we assessed changes in metabolites from admission to 7 days after aneurysm rupture, we are not able to determine the exact mechanisms by which certain metabolites may mitigate muscle atrophy. Nevertheless, this is one of the first studies to better understand the systemic metabolic effects of HPRO and NMES and provides an important foundation for additional studies. Further, the study groups were randomized, there was careful implementation of the study intervention, and metabolites were analyzed with statistical rigor.

## Conclusions

The HPRO + NMES intervention results in a distinction profile of circulating metabolites including a variety of amino acid derivatives. Further larger studies are required to elucidate the mechanisms by which amino acid metabolic intermediates such as N-acetylleucine prevent muscle atrophy and evaluate their potential therapeutic use after aSAH.

## Figures and Tables

**Table 1:** Demographics and intervention characteristics

	All	SOC	HPRO + NMES	*P*-Value*
**N**	24	12	12	
**Age (mean±SD)**	57.9±11.1	55.6±13.3	60.2±8.1	0.323
**Sex [Female (%)]**	16 (66.7)	9 (75)	7 (58)	0.665
**Race [N (%)]**				0.999
**Black**	9 (37.5)	5 (41.7)	4 (33.3)	
**White**	15 (62.5)	7 (58.3)	8 (66.7)	
**HHS**	3.33±0.96	3.3±1.1	3.4±0.9	0.681
**DCI [N (%)]**	6 (25)	3 (25)	3 (25)	1.00
**mRS discharge 0–3 [N (%)]**	8 (33.3)	4 (33.3)	4 (33.3)	1.00
**mRS 3-months 0–3 [N (%)]**	22 (91.7)	10 (83.3)	12 (100)	0.460
**Protein per day (g/kg/d, mean±SD)**	1.21±0.52	0.9±0.37	1.51±0.47	0.0020
**Nitrogen balance (mean±SD)**	−1.29±5.24	−4.74±4.35	1.88±3.86	9.01×10^−4^
**Muscle Volume**				
**Quadricep [%±SD]**	−7.23±7.89	−12.1±6.17	−3.17±6.94	0.0049
**Temporalis [%±SD]**	−11.5±8.59	−16.1±9.07	−6.96±5.19	0.0089

* *P*-Values reflect comparisons between SOC and HPRO + NMES.

Abbreviations: SOC (standard of care), HPRO + NMES (high protein plus neuromuscular electrical stimulation), HHS (Hunt Hess Scale), mRS (modified Rankin Scale), SBBP (Short Performance Physical Battery), ΔSBBP (change in SBBP from admission to 90-days).

**Table 2:** Multivariable Regression Models for Muscle Volume

	Temporalis Volume		Quadricep Volume	
Multivariable Model	AOR (CI)	P	AOR (CI)	P
1. Protein per day	1.13 (1.07, 1.20)	**5.69×10^−4^**	1.13 (1.07, 1.19)	**2.21×10^−4^**
2. Nitrogen balance	1.01 (1.00, 1.02)	**0.0019**	1.01 (1.00, 1.01)	**0.045**
3. N-acetylleucine	1.08 (1.01, 1.16)	**0.038**	1.08 (1.02, 1.15)	**0.012**
4. Quinolinate	1.05 (1.01, 1.09)	**0.018**	1.04 (1.00, 1.07)	**0.040**
5. N-acetylserine	1.18 (1.04, 1.34)	**0.017**	1.13 (1.01, 1.27)	0.055
6. N-acetylisoleucine	1.11 (1.01, 1.22)	**0.048**	1.08 (0.99, 1.18)	0.12
7. β-hydroxyisovaleroylcarnitine	1.07 (1.00, 1.15)	0.059	1.07 (1.01, 1.13)	**0.026**

Abbreviations: AOR, adjusted odds ratio; CI, 95% confidence interval. All models are adjusted for age, sex, and aSAH severity (HHS I-III vs IV-V).

## Non-Standard Abbreviations and Acronyms:

aSAH: aneurysmal subarachnoid hemorrhage
HPRO: high protein diet
NMES: neuromuscular electrical stimulation
sPLS-DA: squared discriminant analysis
SOC: standard of care
IMM: immune-mediated malnutrition
HHS: Hunt Hess score
mFS: modified Fisher Scale
BMI: body mass index
mRS: modified Rankin Scale
PBD: post-bleed day
SPPB: short performance physical battery
EDTA: ethylenediaminetetraacetic acid
UPLC-MS/MS: ultra-high-performance liquid chromatography-tandem mass spectrometry
AUC: area of under the curve
NifTI: Neuroimaging Informatics Technology Initiative
FC: fold change
FDR: false discovered rate
NAL: N-acetylleucine
NAD+: nicotinamide adenine dinucleotide
NMDA: N-methyl-D-aspartate
TBI: traumatic brain injury
AKI: acute kidney injury
